# Aberrant Glycosylation Augments the Immuno-Stimulatory Activities of Soluble Calreticulin

**DOI:** 10.3390/molecules23030523

**Published:** 2018-02-27

**Authors:** Fang-Yuan Gong, Zheng Gong, Cui-Cui Duo, Jun Wang, Chao Hong, Xiao-Ming Gao

**Affiliations:** Institute of Biology and Medical Sciences, Soochow University, Suzhou 215123, Jiangsu Province, China; 20154221101@stu.suda.edu.cn (Z.G.); gfy0308@sina.com (C.-C.D.); jwang79@suda.edu.cn (J.W.) chaohong@suda.edu.cn (C.H.)

**Keywords:** calreticulin eukaryotic, macrophage, glycosylation

## Abstract

Calreticulin (CRT), a luminal resident calcium-binding glycoprotein of the cell, is a tumor-associated antigen involved in tumorigenesis and also an autoantigen targeted by autoantibodies found in patients with various autoimmune diseases. We have previously shown that prokaryotically expressed recombinant murine CRT (rCRT) exhibits strong stimulatory activities against monocytes/macrophages in vitro and potent immunogenicity in vivo, which is partially attributable to self-oligomerization of soluble rCRT. However, even in oligomerized form native CRT (nCRT) isolated from mouse liver is much less active than rCRT, arguing against the possibility that self-oligomerization alone would license potent pro-inflammatory properties to nCRT. Since rCRT differs from nCRT in its lack of glycosylation, we wondered if aberrant glycosylation of eukaryotically expressed CRT (eCRT) would significantly enhance its immunological activity. In the present study, tunicamycin, an *N*-glycosyltransferase inhibitor, was employed to treat CHO cells (CHO-CRT) stably expressing full-length recombinant mouse CRT in secreted form for preparation of aberrantly glycosylated eCRT (tun-eCRT). Our biochemical and immunological analysis results indicate that eCRT produced by CHO-CRT cells is similar to nCRT in terms of glycosylation level, lack of self-oligomerization, relatively poor immunogenicity and weak macrophage-stimulatory activity, while tun-eCRT shows reduced glycosylation yet much enhanced ability to elicit specific humoral responses in mice and TNF-α and nitric oxide production by macrophages in vitro. Given that abberant glycosylation of proteins is a hallmark of cancer cells and also related to the development of autoimmune disorders in humans, our data may provide useful clues for better understanding of potentiating roles of dysregulated glycosylation of molecules such as CRT in tumorigenesis and autoimmunity.

## 1. Introduction

Calreticulin (CRT) is an endoplasmic reticulum (ER) residential glycoprotein playing important roles in maintaining intracellular calcium homeostasis and also facilitating correct folding of major histocompatibility complex (MHC) class I molecules [1,2]. It is also regarded as a tumor-associated antigen because CRT is present on the membrane surface of various tumor cells and soluble CRT can be detected in the body fluid of patients with lung or bladder cancer [1,2,3,4]. The N-domain of CRT has been labeled as “vasostatin” for its ability to facilitate blood vessel formation in cancerous tissues [5]. Additionally, CRT is an important autoantigen targeted by autoantibodies in patients with autoimmune disorders such as rheumatoid arthritis (RA) and systemic lupus erythematosus (SLE) [3]. The angiogenesis activity of CRT may contribute to the joint inflammation of RA [6]. However, detailed molecular mechanisms for roles of CRT in tumorigenesis and autoimmunity so far remain illusive. 

Our earlier studies showed that prokaryotically expressed recombinant murine CRT (rCRT, without the leader and ER-retention sequences) and CRT fragment covering amino acid residues 39-272 (rCRT/39-272, including the N and partial P domains) are capable of inducing strong antibody responses in mice and also macrophage production of tumor necrosis factor-α (TNF-α) and interleukin (IL)-6 in vitro [7,8]. Since TNF-α and IL-6 represent predominant inflammatory cytokines in the pathogenesis of RA and SLE [9,10], our results underlie a strong correlation between serum CRT levels and autoimmune diseases. The pro-inflammatory property of rCRT is partially attributable to its ability to self-oligomerize in solution [8], which is supported by the fact that native CRT (nCRT) isolated from mouse liver exists in monomeric form in solution and is much less active compared with prokaryotically expressed rCRT. There are three Cys in CRT. Cys105 and Cys137 form intramolecular disulfide bonds, while Cys163 is free. Jorgensen et al documented that shielding of the free Cys163 in the N domain is the main reason that nCRT exists mainly in monomeric form under physiological conditions. Under partial unfolding conditions such as high temperature or low PH, however, the free Cys could be exposed and subsequently help CRT oligomerization [11]. Ironically, however, oligomerization of nCRT, induced by high temperature and/or low pH, did not lead to significantly enhanced macrophage-stimulatory activity of the molecule [12]. 

Given that rCRT differs from nCRT in its lack of glycosylation, we asked if aberrant glycosylation of eukaryotically expressed CRT (eCRT) would significantly enhance its immunological activity. It has been documented that murine CRT possesses one *N*-glycosylation site at residue Asn 327 [13,14] and also that CRT glycosylation is susceptible to modulation by extracellular stimuli [15,16]. Additionally, nCRT purified from various tissues carries different glycosylation moieties [13,14]. This study was thus designed to investigate if aberrant glycosylation of eukaryotically expressed CRT (eCRT) could augment its immunological activity. Given that aberrant glycosylation of proteins is a hallmark of cancer cells and also correlated to autoimmune diseases in humans, results from this study may provide useful clues for a better understanding of the dysregulated glycosylation of CRT in tumorigenesis and autoimmunity.

## 2. Results

### 2.1. Expression, Purification and Functional Characterization of eCRT

**Figure 1:**
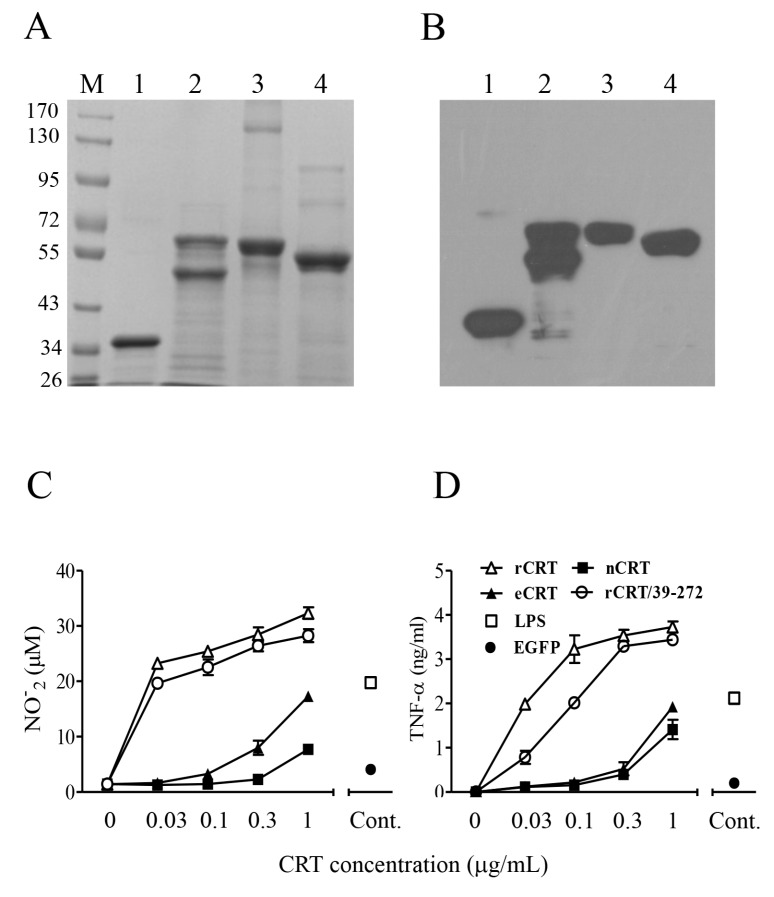
Purification and functional characterization of eCRT. Purified rCRT/39-272, rCRT, eCRT and nCRT (lanes 1–4, respectively) were separated by SDS-PAGE (**A**) and detected by rabbit anti-CRT polyclonal antibodies in Western-blot (**B**). Mouse peritoneal macrophages were stimulated with these CRT preparations of increasing concentrations for 24 h, followed by quantitation of NO (**C**) and TNF-α (**D**) in the culture supernatant using Griess Reagent or ELISA, respectively. Macrophages stimulated with LPS (1 ng/mL) or rEGFP (1 µg/mL) were included as controls.

Murine rCRT and rCRT/39-272, both expressed in *E. coli*, and nCRT from mouse liver were affinity-purified by Ni^+^ columns and ion-exchange columns, respectively, as previously documented [7,8]. Murine CRT gene encoding aa residues 18-412 (less the leader and ER retention sequences) was cloned into expression vector pIRES2 that encodes a secretion leader sequence from pDisplay at the N terminus and 6-histidine (His) tag at the C terminus. The resultant plasmid (pIRES2-eCRT) was transfected into CHO cells for preparation of a cell line (CHO-CRT) stably expressing secreted eCRT, which is affinity-purifiable from the cell culture supernatant by Ni^+^ columns. The purity of fractionated rCRT/39-272, rCRT, nCRT and eCRT was approximately 90% as judged by Coomassie brilliant blue-stained SDS-PAGE, and the protein bands of expected molecular mass were specifically recognized by rabbit anti-mouse CRT polyclonal Abs in Western blotting (Figure 1A,B). Note that partial degradation occurred to rCRT during the purification process (Figure 1A, lane 2), which is in line with our earlier report [8]. The smaller fragment represented rCRT less the C-terminal 26 residues and it was equally active as full-length rCRT in terms of stimulatory effect against macrophages in vitro and immunogenicity in vivo [8]. Both rCRT/39-272 and rCRT showed strong ability to elicit TNF-α and nitric oxide (NO) production by murine macrophages, while eCRT and nCRT were approximately 10 folds less effective than rCRT and rCRT/39-272 in parallel experiments (Figure 1C,D). 

### 2.2. Immunogenicity of eCRT, rCRT and nCRT

**Figure 2:**
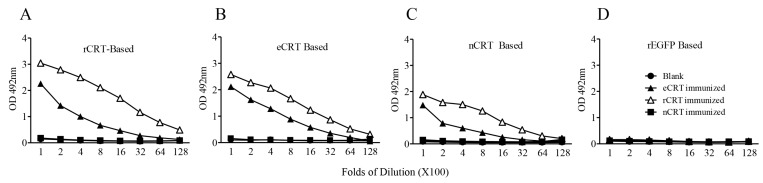
Immunogenicity of eCRT, nCRT and rCRT in mice. SDS-PAGE gel slices containing bands of rCRT, eCRT and nCRT were used as antigens for s.c. immunization of C57/BL6 mice (100 µg/mouse, *n* = 5) and boosted with 50 µg of the same antigen preparations a fortnight later, with blank gel slices as negative control (Blank). The mice were bled 10 days thereafter for sera that were assayed, in triplicate wells, in ELISAs based on rCRT (**A**), eCRT (**B**), nCRT (**C**) or rEGFP (**D**). The detection Ab was HRP-conjugated goat-anti-mouse IgG with OPD as substrate, and the results are expressed as mean OD492 nm ± SD of three replicates. These are representatives of three independent experiments using proteins from different bulks of purifications.

We next compared the ability of eCRT, nCRT and rCRT to induce antibody responses in mice. To avoid interference of residual contaminants in the antigen preparations, eCRT, nCRT and rCRT were run in SDS-PAGE gels, then the dominant protein bands of expected molecular mass were cutout and the resultant gel slices used as immunogens in subsequent animal immunization experiments. Groups of C57BL/6 mice were subcutaneously immunized with the antigens, followed by collection of serum samples 10 days thereafter for titration of CRT-specific IgG in ELISAs. Figure 2 shows that antisera from mice immunized with rCRT strongly recognized rCRT, eCRT as well as nCRT. Antisera from eCRT-immunized mice were also able to bind these CRT preparations, albeit with approximately 2–10 folds lower titers compared with that of rCRT-induced antisera. Serum samples from nCRT-immunized, or unimmunized, mice did not show any measurable binding to CRTs, and none of the antisera from CRT-immunized mice recognized recombinant green fluorescence protein (rEGFP) expressed in *E. coli* (Figure 2). 

### 2.3. Biochemical Comparison of eCRT and nCRT

**Figure 3:**
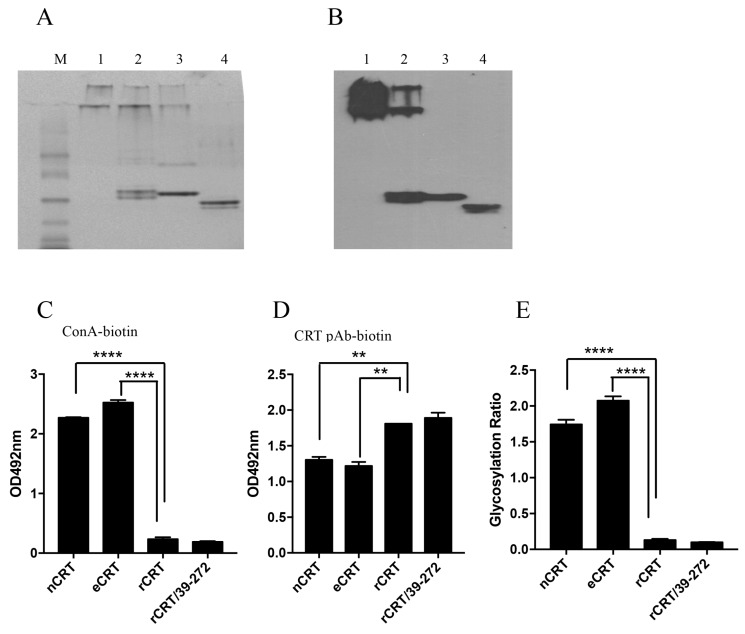
Biochemical properties of eCRT and rCRT. Samples of rCRT/39-272, rCRT, eCRT and nCRT (lanes 1–4, respectively) were compared in Coomassie brilliant blue-stained native PAGE (**A**), followed by WB using rabbit anti-CRT polyclonal antibody for detection (**B**). The secondary Ab was HRP labeled goat-anti-rabbit IgG, with OPD as substrate. ELISA plates were pre-coated with nCRT, eCRT, rCRT or rCRT/39-272 followed by ELISAs using ConA-biotin (**C**) or biotin-conjugated mouse anti-CRT polyclonal antibody (**D**) for detection. The glycosylation ratios of the CRT preparations were calculated as OD_ConA_/OD_pAb_ (**E**). Results are expressed as mean ± SD of three replicates. **** *p* < 0.0001; ** *p* < 0.01. These are representatives of 3 independent experiments.

Based on our previous results that self-oligomerization could significantly enhance immunological activity of CRT [8], it was reasonable to ask if the enhanced immunogenicity of eCRT over nCRT was due to self-oligomerazation in solution. Native PAGE followed by Western blotting was performed to compare the oligomerization status of the CRT preparations. As expected, rCRT and rCRT/39-272 existed mostly (approximately over 90% and 60%, respectively) as oligomers in solution at neutral pH. However, eCRT and nCRT existed only in monomeric form in the same conditions (Figure 3A,B). It has previously been documented that calcium depletion as well as N- and C-terminal truncations promotes CRT oligomerization [17]. Since rCRT and eCRT share the same N- and C-terminal truncations and all CRT preparations were stocked in calcium-free solution, our results imply that glycosylation status may play an important role in controlling self-oligomerization of CRT. 

To semi-quantitatively assess the glycosylation levels of CRT preparations, we employed biotinylated ConA, which can bind protein-conjugated oligosaccharide moieties with high affinity, as a detecting agent. ELISA plates were pre-coated with nCRT, eCRT, rCRT or rCRT/39-272, followed by biotin-ConA and then streptavidin-HRP with OPD as substrate. As shown in Figure 3C, ConA binding to eCRT was similarly strong as to nCRT, but there was hardly any ConA binding to rCRT and rCRT/39-272. Even CRT protein coating across the groups was confirmed by strong and specific binding of anti-CRT Abs to all pre-coated wells (Figure 3D). Clearly, relative glycosylation ratios in eukaryotical CRT (eCRT and nCRT) were significantly higher than that in the prokaryotical CRT (rCRT and rCRT/39-272) (Figure 3E). 

### 2.4. Dys-glycosylation of eCRT Correlates to Enhanced Immunological Activity

**Figure 4:**
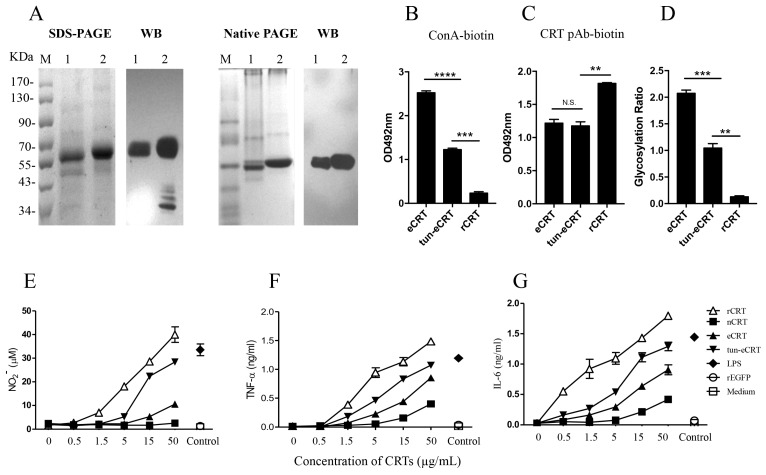
The effect of de-glycosylation on the immune-stimulatory effect of eCRT. Confluent CHO-CRT cells were cultured in serum-free medium in the presence of 5 µg/mL tunicamycin for 48 h. The supernatant was then harvested for purification of tun-eCRT using Ni-columns. Samples of tun-eCRT (lane 1) and eCRT (lane 2) were separated by SDS-PAGE or native PAGE and detected by WB using rabbit anti-CRT polyclonal antibody (**A**). The glycosylation levels of eCRT, tun-eCRT and rCRT were compared in biotin-ConA binding assays (**B**) with CRT-Based ELISAs as even protein coating control (**C**). The glycosylation ratios of the CRT preparations were determined by OD_ConA_/OD_pAb_ (**D**). Mouse peritoneal macrophages were stimulated with increasing concentrations of eCRT, tun-eCRT, nCRT or rCRT for 24 h, NO (**E**), TNF-α (**F**) and IL-6 (**G**) in the culture supernatant were quantitated by Griess Reagent and ELISAs, respectively. Results are expressed as mean ± SD of three replicates. **** *p* < 0.0001;*** *p* < 0.001; ** *p* < 0.01. These are representatives of three independent experiments.

**Figure 5:**
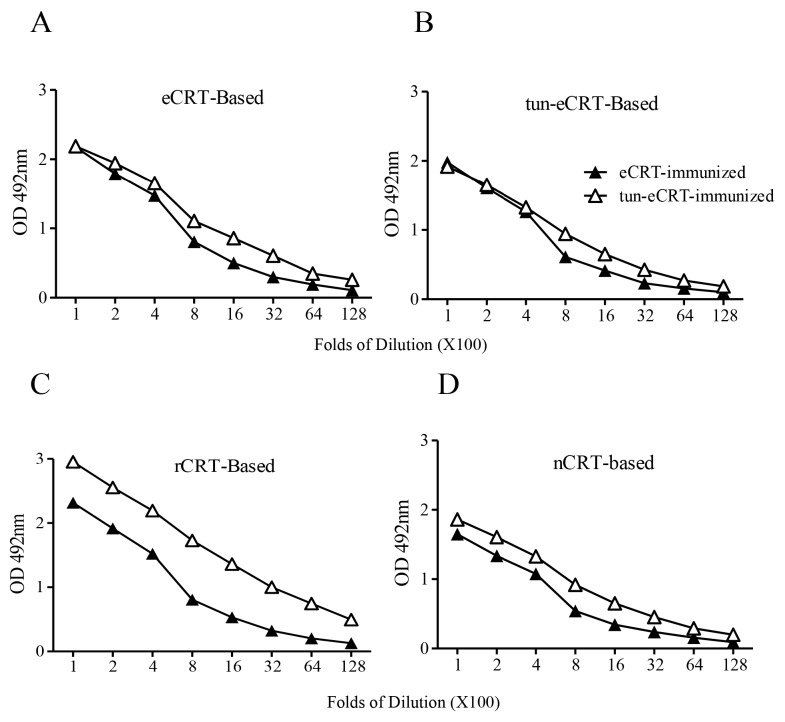
Comparison of immunogenicity of eCRT and tun-eCRT. Coomassie-blue stained SDS-PAGE gel slices containing eCRT or tun-eCRT were used immunogens for s.c. immunization of C57/BL6 mice (*n* = 5). Immunized mice were boosted with the same antigens with fortnight intervals. Mice were bled 10 days thereafter for detection of antigen-specific IgG in ELISAs based on eCRT (**A**), tun-eCRT (**B**), rCRT (**C**) or nCRT (**D**). Results are expressed as mean ± SD of three replicates. These are representatives of 3 independent experiments.

In order to further investigate the effect of aberrant glycosylation on the immunological activity of eCRT, tunicamycin, an *N*-glycosyltransferase inhibitor [18], was employed to treat CHO-CRT cells for preparation of dys-glycosylated eCRT (tun-eCRT). The molecular mass of affinity purified tun-eCRT was slightly lower than eCRT as judged by SDS-PAGE (Figure 4A). The native-PAGE results confirmed that, similar to rCRT, tun-eCRT exists mainly in monomeric rather than oligomeric form. ConA ELISAs further revealed that the level of glycosylation of tun-eCRT was significantly lower than that of eCRT but much higher than that of rCRT (Figure 4B–D). Note that rabbit anti-mouse CRT polycolnal antibodies could not distinguish eCRT from tun-eCRT (Figure 4C), implying that aberrant glycosylation of tun-eCRT did not drastically affect its antigenicity. More importantly, tun-eCRT was apparently more effective than eCRT and nCRT in eliciting macrophage production of NO, TNF-α and IL-6 in vitro (Figure 4E–G). Antisera from mice immunized with tun-eCRT showed significantly higher IgG titers against CRT in ELISAs (Figure 5). Together these results show that aberrant glycosylation enhances pro-inflammatory activity and immunogenicity without affecting monomeric status of eCRT.

## 3. Discussion

CRT is susceptible to various post-translation modifications with important functional consequences. It has been reported that CRT can be phosphorylated by PKC [19] or c-src [20], which enables the molecule to stabilize mRNA in the cell. Arginylation of CRT, which causes a greater degree of dimerization and oligomerization of the molecule [21], makes the cell more susceptible to apoptosis [22]. Glycosylation is another important post-translation modification of CRT, which can impact its cellular redistribution [23]. CRT glycosylation changes rapidly and dynamically in response to extracellular stimuli such as heat shock and cold stress [15,24]. PKC activation could also abolish CRT *N*-glycosylation but increase *O*-linked-*N*-acetylglucosamine modification [25]. Furthermore, nCRT isolated from different sources, such as rat and bovine liver [26,27], bovine brain [28], hamster ovary [15], or human myeloid cells [29] appears to possess diverse glycosylation moieties. However, contribution of the glycosylation moieties of CRT to its oligomerization under different conditions has not been thoroughly investigated. 

Here we compared eCRT and tun-eCRT for glycosylation level, self-oligomerization, macrophage-stimulatory activity in vitro and also immunogenicity in vivo. Our results indicate that tunicamycin-induced dys-glycosylation of eCRT significantly enhanced the immune-stimulatory activity and immunogenicity without affecting the monomeric status of the molecule. The lack of immunogenicity of nCRT in mice can be explained by self-tolerance of the host to tissue antigens. Yet eCRT is identical to nCRT in terms of amino acid sequence, the apparently stronger immunogenicity of eCRT than nCRT has to be explained by either incorrect folding, increased self-oligomerization or aberrant post-translation modifications such as glycosylation. Although the extraordinarily strong immune-stimulatory activity of prokaryotically expressed rCRT was attributable to its self-oligomerization in solution [8], eCRT as well as tun-eCRT did not seem to self-oligomerize in solution (Figure 3), thereby arguing against any substantial role of oligomerization in licensing potent immunological activity to eukaryotical CRT. We propose that dys-glycosylation rather than self-oligomerization is a more important factor rendering eCRT immunological activities. Although the tunicamycin could inhibit glycosylation of recombinant CRT in CHO, tunicamycin treatment is not for thoroughly de-glycosylation and might induce other effects on CHO cells thus affecting CRT post-translational modification. An alternative system for preparation of de-glycosylated CRT would be CHO-Lec1 cells that are deficient in *N*-acetylglucosaminyltransferase I (GlcNAc-TI) and do not synthesize *N*-glycans. The only possible site for glycosylation of murin CRT is Asn 327, thus murine CRT with amino acid 327 mutation could also be used for confirmation the contribution of glycosylation to immune-stimulatory activity of CRT. Both lines of work are currently in progress in this laboratory. 

Higher levels of serum CRT positively correlate with RA, SLE and certain cancers in humans [1,2,3,4,7]. Ding et al. also reported that extracellular CRT may promote angiogenesis via a NO-mediated pathway in RA [6]. This is in line with our finding that tun-eCRT is able to elicit inflammatory mediator (including NO) production by macrophages. Aberrant protein glycosylation has been associated with tumors as well as various autoimmune syndromes, such as RA and SLE [30]. Defect in protein *N*-glycosylation pathway is a genetic cause of autoimmune disease [31]. A bioinformatical approach by Szabo and colleagues predicated substantial impact of aberrant glycosylation of certain proteins on T cell autoimmunity [32]. These data point to a possibility that CRT from patients may possess potent pro-inflammatory (immunostimulatory) or angiogenesis activities as does tun-eCRT. Therefore, the glycosylation and immunological characteristics of CRT in patient sera or in cancerous tissues merits further detailed investigation.

## 4. Materials and Methods

### 4.1. Construction of Plasmid pIRES-eCRT

Total cellular RNA was extracted from mouse tumor cell line EL4 (ATCC) using TRIzol (Invitrogen, Carlsbad, CA, USA) and then reverse transcribed with Oligo-dT using a SuperScript II reverse transcriptase kit (Invitrogen, Carlsbad, CA, USA) according to the manufacturer’s instructions. The resultant cDNA was employed as template for amplification of the gene encoding CRT fragment 18-412 by PCR using forward primer 5′-ATT**CCGCGG**TGACCCTGCCATCTATTTCAAA-3′ with a Sac II restriction site and reverse primer 5′-ATATA**GTCGAC**CTAG*TGGTGGTGGTGGTGGTGGTGG*TTGGCCAGGGGATTCTTCC-3′ with a Sal I restriction site. The PCR product (encoding the target sequence with a C-terminal 6X polyhistidine tag) was cloned into expression vector pDisplay (Invitrogen, Carlsbad, CA, USA). The gene encoding CRT/18-242 with N-terminus leader sequence of pDisplay and C-terminus His tag was finally cloned into expression vector pIRES2 using a Xho I and a Sal I restriction sites. 

### 4.2. Expression and Purification of CRT

For preparation of secreted eCRT, CHO cells were transfected with plasmid pIRES-eCRT using lipofectamine 2000 (Invitrogen, Carlsbad, CA, USA) according to the manufacturer’s specifications. Stable transfections (CHO-eCRT) were selected in G418 (1 mg/mL, Sigma, St. Louis, MO, USA) for two weeks. Transfected CHO cells were grown in 10 cm dishes until confluent, culture medium was then removed and changed into FBS-free DMEM (Hyclone, Logan, UT, USA) after being washed with warm PBS. Cells were further incubated for 48 h (5% CO_2_, 37 °C) and supernatant harvested and depleted cell debris by centrifugation, followed by clearing using 0.45 µM filter (Millipore, Billerica, MA, USA). For preparation of tun-eCRT, CHO-CRT cells were cultured in FBS-free DMEM in the presence of tunicamycine (5 µg/mL, Sigma, St. Louis, MO, USA) for 48 h. The secreted recombinant eCRT was purified from the supernatant using Ni-column (GE, Little Chalfont, Buckinghamshire, UK) following the manufacturers’ instructions. Briefly, the supernatant was loaded on the column, washed with buffer containing 20 mM Tris-HCl, 0.5 M NaCl, 20 mM Imidazole, pH 7.9. The proteins were eluted with wash buffer containing 60 mM Imidazole and dialyzed against PBS, pH 7.9, for 16 h. Prokaryotical rCRT and rEGFP were expressed and purified as previously described [7,8]. Briefly, *E. coli* BL21 (DE3) (Stratagene, La Jolla, CA, USA) containing PET28a-EGFP or PET28a-CRT were grown to log phage at cell concentration of OD600 was 0.6–0.8 in medium with 30 µg/mL kanamycin at 37 °C, and protein expression was induced by adding 0.1 mmol/L isopropylthio-β-d-galactoside (IPTG) in LB medium for further incubation at 30 °C for 5 h. The bacterial lysate (with protease inhibitor PMSF) was loaded on the column, washed with buffer containing 20 mM Tris-HCl, 0.5 M NaCl, 20 mM Imidazole, pH 7.9. The proteins were eluted with wash buffer containing 300 mM Imidazole. nCRT was purified as previously described using ion-exchange column [8]. All the proteins were desalted into PBS and concentrated by ultra-centrifugate tube. Concentration of protein was quantified using Pierce BCA Protein Assay Kit (Pierce, Invitrogen). The proteins were treated by polymyxin B-agarose (GE Health, Little Chalfont, Buckinghamshire, UK) for three times before being used and endotoxin acitivity of proteins were both less than 0.01 EU/µg protein assessed by the limulus amebocyte assay.

### 4.3. SDS-PAGE, Native PAGE and Western Blotting

For SDS-PAGE, loading buffer with 1% SDS and 2-ME was used. For native PAGE, the loading buffer did not contain any detergent or reducing agents. The gels were stained with Coomassie brilliant blue or transferred to PVDF membranes (Millipore, Billerica, MA, USA). Immunoblots were blocked with 5% non-fat milk (*w*/*v*) in TBS buffer for 1 h at room temperature then incubated with primary rabbit antibody against CRT (Pierce, 1:1000) over-night. Immunoreactive bands were detected using HRP-conjugated secondary antibodies with the western lighting chemiluminescence plus reagent (BD Pharmingen, San Jose, CA, USA).

### 4.4. Isolation and Culture of Mouse Peritoneal Macrophages

For preparation of murine macrophages, C57BL/6 mice (female, 6–8 weeks) were injected intraperitoneally with 1 mL 3% thioglycollate (BD Pharmingen, San Jose, CA, USA). Peritoneal cells were harvested by peritoneal lavage with RPMI-1640 72 h after injection and seeded in 96-well tissue culture plates. After removing non-adherent cells, the remaining cells were 90% positive for macrophage surface marker F4/80, as determined by FACS analysis. Macrophages were cultured in complete R10 medium: RPMI-1640 supplemented with 10% (*v*/*v*) fetal bovine serum (Hyclone, Logan, UT, USA), penicillin/streptomycin (100 U/mL), l-glutamine (2 mM), and β-ME (5 × 10^−5^ M). After stimulation with eCRT, tun-eCRT, rCRT and EGFP for 24 h, TNF-α and IL-6 in supernatant was determined by ELISA kit (Biolegend, San Diego, CA, USA) according to manufacturer’s instructions. NO in supernatant was determined by NO detection Kit (Promega, Fitchburg, WI, USA). All procedures were approved by Ethic Committee of Soochow University (IACUC-01326)

### 4.5. Immunization of Mice and Protein Based ELISA

CRT preparations (100 µg per gel) were separated in SDS-PAGE 10% gels, gel slices containing expected CRT bands were cutout with a razorblade and then frozen in liquid nitrogen, minced and then re-suspended in PBS. Groups of C57BL/6 mice (5 per group) were subcutaneously immunized with 100 µL CRT and boosted twice a fortnight later interval with the same antigens. The immunized mice were bled 7 days after the immunization for assessment of serum titers against plate-coated CRT in ELISAs.

For CRT-based ELISAs, 2 µg/mL protein was coated on plate in carbonate buffer (pH 9.6) and subsequently incubated with blocking solution (2% BSA in PBS) for 2 h at 37 °C. The wells were washed five times with PBS containing 0.05% Tween 20 (PBS-T) and then 100 µL of diluted immunized mouse sera in PBS were added in triplicates and incubated for 2 h at 37 °C. After five washes with PBS-T, the plates were incubated with HRP-labeled goat anti-mouse IgM or IgG Abs (Southern Biotech) for 1 h at 37 °C. The reaction was developed with 100 µL of *O*-phenylenediamine (OPD) (Sigma-Aldrich, St. Louis, MO, USA) for 5 min and stopped with 50 µL of 2 M H_2_SO_4_. OD was measured at 492 nm in an ELISA spectrophotometer (Bio-Tek, Winooski, VT, USA). 

### 4.6. Detection of N-glycosylation in CRTs

Lectin blot analysis was essentially the same as ELISAs as previously described [25]. ELISA plates were coated with CRTs (0.2 µg/mL) in carbonate buffer (pH 9.6) and subsequently incubated with blocking solution (2% BSA in PBS, *w*/*v*) for 2 h at 37 °C. The wells were washed five times with PBS-T and then incubated with 1 µg/mL biotinylated Con A (Sigma, St. Louis, MO, USA) for 1 h at 37 °C. *N*-glycosylated proteins were visualized by incubation with HRP-conjugated streptavidin and developed with 100 µL of *O*-phenylenediamine (OPD) (Sigma-Aldrich, St. Louis, MO, USA) for 5 min and stopped with 50 µL of 2 M H_2_SO_4_. OD was measured at 492 nm in an ELISA spectrophotometer. No detectable ConA binding was observed when Peptide *N*-glycosidase F (PNGase F)-treated eCRT and tun-eCRT were analyzed in this system. Negative controls were also obtained by incubating wells with biotinylated ConA in the presence of 300 mM free glucose prior to visualization with HRP-conjugated streptavidin. 

### 4.7. Statistical Analysis

All experiments were repeated at least three times. Statistical analysis was performed using the independent samples *t* test or one-way ANOVA and Tukey’s post hoc tests. Differences were considered statistically significant at *p* < 0.05.
